# MicroRNA Alterations in Chronic Traumatic Encephalopathy and Amyotrophic Lateral Sclerosis

**DOI:** 10.3389/fnins.2022.855096

**Published:** 2022-05-19

**Authors:** Marcela Alvia, Nurgul Aytan, Keith R. Spencer, Zachariah W. Foster, Nazifa Abdul Rauf, Latease Guilderson, Ian Robey, James G. Averill, Sean E. Walker, Victor E. Alvarez, Bertrand R. Huber, Rebecca Mathais, Kerry A. Cormier, Raymond Nicks, Morgan Pothast, Adam Labadorf, Filisia Agus, Michael L. Alosco, Jesse Mez, Neil W. Kowall, Ann C. McKee, Christopher B. Brady, Thor D. Stein

**Affiliations:** ^1^Boston University Alzheimer’s Disease Research Center, Boston University CTE Center, Boston University School of Medicine, Boston, MA, United States; ^2^Department of Neurology, Boston University School of Medicine, Boston, MA, United States; ^3^VA Boston Healthcare System, Boston, MA, United States; ^4^Southern Arizona VA Healthcare System, Tucson, AZ, United States; ^5^Department of Veterans Affairs Medical Center, Bedford, MA, United States; ^6^Department of Pathology and Laboratory Medicine, Boston University School of Medicine, Boston, MA, United States

**Keywords:** chronic traumatic encephalopathy, amyotrophic lateral sclerosis, microRNA, contact sports, p-tau, TDP-43, prefrontal cortex

## Abstract

Repetitive head impacts (RHI) and traumatic brain injuries are risk factors for the neurodegenerative diseases chronic traumatic encephalopathy (CTE) and amyotrophic lateral sclerosis (ALS). ALS and CTE are distinct disorders, yet in some instances, share pathology, affect similar brain regions, and occur together. The pathways involved and biomarkers for diagnosis of both diseases are largely unknown. MicroRNAs (miRNAs) involved in gene regulation may be altered in neurodegeneration and be useful as stable biomarkers. Thus, we set out to determine associations between miRNA levels and disease state within the prefrontal cortex in a group of brain donors with CTE, ALS, CTE + ALS and controls. Of 47 miRNAs previously implicated in neurological disease and tested here, 28 (60%) were significantly different between pathology groups. Of these, 21 (75%) were upregulated in both ALS and CTE, including miRNAs involved in inflammatory, apoptotic, and cell growth/differentiation pathways. The most significant change occurred in miR-10b, which was significantly increased in ALS, but not CTE or CTE + ALS. Overall, we found patterns of miRNA expression that are common and unique to CTE and ALS and that suggest shared and distinct mechanisms of pathogenesis.

## Introduction

Chronic traumatic encephalopathy (CTE) is a neurodegenerative disease associated with years exposure to repetitive head impacts (RHI). Chronic traumatic encephalopathy has been reported in a wide variety of RHI exposures, including contact sports such as American football, boxing, hockey, and rugby as well as from military blast injuries. Clinical symptoms may involve multiple domains, including mood, behavior, and cognitive functions (Katz et al., 2021). In some cases, motor symptoms can emerge in the form of parkinsonism (Adams et al., 2018) or motor neuron disease/amyotrophic lateral sclerosis (ALS) (McKee et al., 2009). Amyotrophic lateral sclerosis is four times more frequent in National Football League players (Lehman et al., 2012; Daneshvar et al., 2021) and is found within ∼6% of contact sports athletes with CTE (Mez et al., 2017). Microscopically, the hallmark of CTE involves phosphorylated tau (p-tau) neurofibrillary tangles (NFTs) that accumulate within neurons and neuronal processes in the cerebral cortex, preferentially at sulcal depths and around blood vessels. TDP-43 is present in approximately half of low stage (stage I and II) CTE and first appears within the CTE p-tau lesions at the sulcal depths of the frontal cortex (Danielsen et al., 2017). In high stage (stage III and IV) CTE, TDP-43 pathology is more frequent and involves additional brain regions (McKee et al., 2013).

Amyotrophic lateral sclerosis (ALS) is characterized by progressive degeneration of motor neurons within the motor cortex of the brain (upper motor neurons) and spinal cord (lower motor neurons). Symptoms typically manifest in one region of the body and progress to paralysis, respiratory failure, and eventual death. In most sporadic cases, pTDP-43 inclusions are present within motor neurons and variably in other regions of the brain. The disease course tends to be rapid with death occurring in 2 to 5 years. Both genetic and environmental factors are linked to the etiology of ALS (Saez-Atienzar et al., 2021). A 2007 study found that a diagnosis of ALS was 11-fold higher in those with multiple head injuries within 10 years than in those with no head injuries (H. Chen et al., 2007; Schmidt et al., 2010).

Chronic traumatic encephalopathy (CTE) with TDP-43 proteinopathy and ALS was first reported in contact sport athletes, including 2 former NFL athletes and one professional boxer (McKee et al., 2010) as well as a young soccer player (McKee et al., 2014). In a study done on the military cohort of the Department of Veterans Affairs Biorepository Brain Bank 5.8% of those with ALS were also comorbid with CTE. These comorbid subjects were more likely to have a history of traumatic brain injury (TBI). Clinically, they were more likely to have a bulbar onset and mood and behavioral alterations (Moszczynski et al., 2018; Walt et al., 2018).

MicroRNAs (miRNAs) are small non-coding strands of RNA of approximately 22 base pairs that are involved in regulating translation of messenger RNA. They target mRNA at the 3′ UTR and may either silence their translation or degrade them (O’Brien et al., 2018). MiRNAs are fairly new in the biomarker field and several studies have been performed that describe that their fluctuations in relation to diseases such as ALS and Alzheimer’s disease (Cheng et al., 2015; Mez et al., 2017; Miya Shaik et al., 2018; Ricci et al., 2018; Dewan and Traynor, 2021; Magen et al., 2021). Their putative involvement in CTE is thus far unknown.

The overlap in CTE and ALS pathologies and risk factors suggests they may share common disease mechanisms, yet the pathways of neurodegeneration might be sufficiently divergent to allow biomarker distinctions and diagnosis during life. Here we set out to determine whether miRNA levels were altered in the prefrontal cortex of participants with CTE, ALS, and comorbid CTE + ALS compared to controls. We hypothesized that individual miRNAs would be differentially regulated in each disease and that some miRNAs would be shared by CTE and ALS.

## Materials and Methods

### Participants and Pathological Groups

Brain donors were selected from the Department of Veterans Affairs Biorepository Brain Bank (Brady et al., 2013) and the Understanding Neurology Injury and Traumatic Encephalopathy (UNITE) study brain bank (Mez et al., 2015, 2017). All consents for research participation and brain donation were provided by next of kin. Institutional Review Boards of the Boston and Bedford VA Healthcare Systems and Boston University Medical Center approved the relevant study protocols.

All brains were examined by neuropathologists (TS, AM, BH, VA) with no knowledge of the clinical data. Diagnoses were made using previously reported protocols and well-established criteria (Mez et al., 2015). The diagnosis of ALS required degeneration of upper and lower motor neurons with degeneration of lateral and ventral corticospinal tracts of the spinal cord and loss of anterior horn cells from cervical, thoracic and lumbar spinal cord with gliosis (Mackenzie et al., 2010). Chronic traumatic encephalopathy was diagnosed using established National Institute of Neurological Disorders and Stroke, NIBIB consensus criteria (McKee et al., 2016; Bieniek et al., 2021) and the McKee staging system (McKee et al., 2013; Alosco et al., 2020).

Brain donors were age and sex (all men) matched, had no other neurodegenerative disease co-morbidities and CTE cases were selected to include all 4 stages. The groups included 16 participants with CTE, 12 with CTE and ALS (CTE + ALS), and 2 controls from the UNITE brain bank (Mez et al., 2015). Fourteen participants with ALS, 9 with CTE + ALS, and 5 controls were selected based on matching diagnosis, age, and sex from the Department of VABBB (Brady et al., 2013). An additional 13 controls were included from the VA National Post-Traumatic Stress Disorder brain bank (Friedman et al., 2017). Controls were without a clinical neurodegenerative disease at post mortem examination. Overall, there were 71 participants, 16 in the CTE group, 21 in the CTE + ALS group, 14 in the ALS group, and 20 participants in the control group (Table 1). There was no significant difference in the age at death or RIN values between the groups.

**TABLE 1:** Variation in pathological group demographics.

	Control *n* = 20	ALS *n* = 14	CTE *n* = 16	CTE+ALS *n* = 21
Age (years)	53.6 (2.5)	59.1 (1.4)	64.9 (3.2)	59.1 (3.4)
Age range (years)	39–70	48–64	34–89	29–87
CTE stage	N/A	N/A	2.56 (0.26)	2.62 (0.2)
RIN	6.8 (0.26)	6.93 (1.4)	6.56 (0.32)	7.48 (0.29)

*Data are expressed as mean (SEM). Amyotrophic lateral sclerosis (ALS), chronic traumatic encephalopathy (CTE).*

### MiRNA Selection

A custom miRNA plate (*Applied BioSystems, Waltham MA*) was designed to include 47 targets previously implicated in human neurodegenerative diseases including Alzheimer disease, ALS, Multiple Sclerosis and Huntington’s disease as determined by PubMed search in May 2019 (Supplementary Tables 1, 2). This study is the first to examine miRNA levels in CTE. MiRNA pathways were determined via Pubmed searches conducted in December 2019 using terms including each miRNA name, Alzheimer Disease, ALS, Huntington’s disease, TBI, multiple sclerosis, Parkinson’s disease, inflammatory, cell death, apoptosis, cell growth, cell proliferation, development, human brain. Each individual miRNA may be involved in multiple different processes, and there is likely overlap between involved pathways.

### Samples and MiRNA Extraction

Whole brain and spinal cord were half frozen and half fixed for complete neuropathological workup as described previously (Brady et al., 2013; Mez et al., 2015). miRNAs were measured within frozen dorsolateral prefrontal cortex gray matter. This region was chosen because it is affected in both diseases and has been utilized in previous studies of gene expression in neurodegenerative diseases (Labadorf et al., 2018).

Approximately 30 mg of frozen prefrontal cortex was homogenized over wet ice by hand using thioglyecrol provided by the *Maxwell RSC miRNA kit* (Promega, Madison WI*).* From this same kit the homogenized tissue was then processed with lysis buffer, DNase and proteinase K solutions. The solution was then inserted into a ready-made cartridge from the kit with all the reagents needed for extraction. MiRNA was extracted and eluted using the *Maxwell 16 Instrument* (Promega).

### Quantitative Real Time Polymerase Chain Reaction

Samples were diluted to 5ng/μl and transcribed into cDNA using a *Taqman Advanced MiRNA cDNA Synthesis Kit from Applied BioSciences*. The cDNA underwent an additional amplification step to increase yields of unstable miRNAs (*MiR-Amp*). Samples were diluted 1:10 and loaded onto qPCR plates with *Taqman Fast Advanced Master Mix*. Each sample received 2 qPCR runs using the *StepOnePlus Real Time polymerase chain reaction (PCR) System* (Applied Biosystems, Foster City, CA), including one to evaluate for U6 a small non-coding spliceosome RNA that is a common endogenous control (Campos-Melo et al., 2013). The next qPCR run was with the custom miRNA plates with primers for selected targets. Samples were tested in duplicate.

### Statistical Analysis

Targets that were successfully amplified had their ΔCT calculated using U6 endogenous control values. All statistics and graphs were generated using GraphPad Prism. Outliers were excluded using the ROUT method set to 0.1%, which resulted in the exclusion of miR-15a-5p from one CTE + ALS sample. Significant changes in each miRNA ΔCT were determined between experimental groups and controls using ANOVA with Dunnett’s multiple comparison testing. In order to further account for the multiple miRNAs tested, a Bonferroni correction of α-value (0.05) divided by the number of successfully amplified miRNA (38) was applied to give a cut-off p-value of 0.00132. For the purposes of graphing the relative change was calculated using the 2^–ΔΔ*CT*^ method (Livak and Schmittgen, 2001).

## Results

**FIGURE 1:**
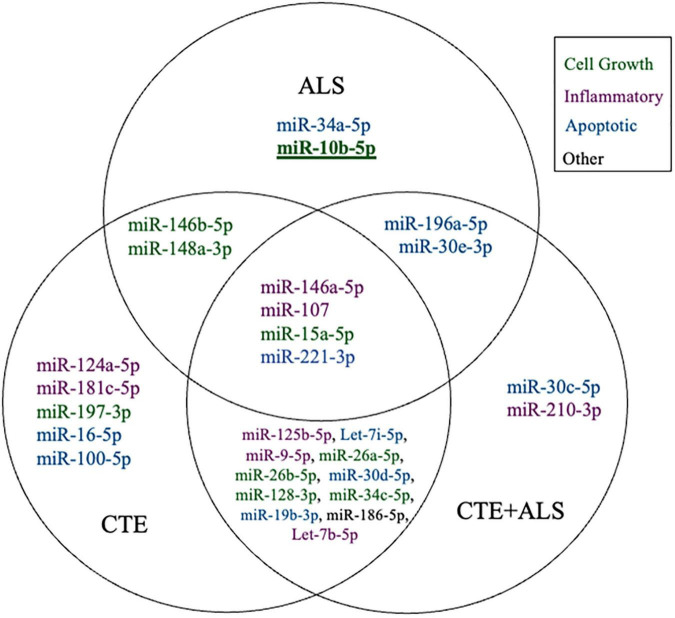
Venn Diagram showing distinct and overlapping significantly altered miRNA within CTE, ALS, and CTE + ALS compared to controls. Bold and underline indicates a p-value at or below the Bonferroni number of 0.0013. Purple indicates miRNAs involved in inflammatory pathways; green indicates cell growth; blue indicates apoptotic; and black indicates miRNA that did not fit in any group.

A total of 38 of the 47 targets were successfully amplified, indicating reliable expression in the human prefrontal cortex. Of those 38 miRNAs, 28 showed a significant difference in ΔCT values across pathology groups using ANOVA (Table 2). Figure 1 shows the distribution and overlap of upregulated miRNA across pathology groups.

**TABLE 2:** Changes in miRNA expression between pathological groups.

MicroRNA	Control	CTE		ALS		CTE+ALS	
	Δ CT	Δ CT	P-value	Δ CT	P-value	Δ CT	P-value
miR-107	–0.07	**−1.48**	**0.0100**	**−1.31**	**0.0337**	**−1.18**	**0.0342**
miR-181c-5p	–2.23	**−3.40**	**0.0489**	–2.89	0.4215	–2.99	0.2298
miR-34c-5p	6.60	**5.18**	**0.0292**	5.35	0.0765	**5.31**	**0.0331**
let-7b-5p	0.02	**−1.43**	**0.0128**	–1.18	0.0591	**−1.23**	**0.0226**
miR-9-5p	–4.78	**−6.06**	**0.0148**	–5.76	0.0971	**−5.94**	**0.0179**
miR-125b-5p	–5.17	**−6.62**	**0.0098**	–6.23	0.0948	**−6.29**	**0.0387**
miR-210-3p	3.54	2.45	0.0747	2.42	0.0789	**2.45**	**0.0491**
miR-124-3p	–6.43	**−7.49**	**0.0493**	–7.01	0.4540	–7.22	0.1439
let-7d-5p	–1.07	–2.03	0.1351	–2.12	0.1112	–1.68	0.4122
miR-146b-5p	1.47	**0.32**	**0.0181**	**0.29**	**0.0184**	0.89	0.3034
miR-197-3p	–6.97	**−8.02**	**0.0444**	–7.89	0.1066	–7.78	0.1181
miR-148a-3p	1.59	**0.52**	**0.0403**	**0.40**	**0.0262**	0.64	0.0533
miR-26b-5p	–3.99	**−5.46**	**0.0069**	–4.99	0.1147	**−5.27**	**0.0129**
miR-26a-5p	–3.81	**−5.36**	**0.0029**	–4.72	0.1395	**−5.04**	**0.0125**
miR-128-3p	–4.35	**−5.79**	**0.0099**	–5.41	0.0952	**−5.45**	**0.0428**
miR-23a-3p	–2.89	–3.90	0.0849	–3.73	0.2038	–3.69	0.1693
miR-34a-5p	–1.50	–5.51	0.0992	**−2.73**	**0.0418**	–2.56	0.0552
miR-100-5p	0.68	**−0.66**	**0.0271**	–0.24	0.2071	–0.26	0.1260
miR-16-5p	–1.34	**−2.72**	**0.0138**	–2.40	0.0920	–2.29	0.0934
miR-19b-3p	0.039	**−1.17**	**0.0333**	–0.92	0.1358	**−1.11**	**0.0289**
miR-30d-5p	–1.09	**−2.13**	**0.0495**	–1.93	0.1535	**−2.25**	**0.0142**
miR-30e-5p	–4.30	–5.35	0.0881	**−5.57**	**0.0380**	**−5.78**	**0.0044**
let-7i-5p	–4.38	**−5.80**	**0.0173**	–5.48	0.1035	**−5.69**	**0.0199**
miR-15a-5p	–2.02	**−3.31**	**0.0161**	**−3.36**	**0.0161**	**−3.08**	**0.0399**
miR-146a-5p	0.44	**−0.69**	**0.0450**	**−0.93**	**0.0153**	**−0.68**	**0.0300**
miR-30c-5p	–4.78	–5.78	0.0553	–5.81	0.0581	**−5.75**	**0.0434**
miR-196a-5p	2.14	0.95	0.0866	**0.68**	**0.0265**	**0.67**	**0.0171**
miR-186	2.40	**0.96**	**0.0112**	1.44	0.1522	**1.30**	**0.0475**
miR-30a-5p	–4.40	–4.81	0.8045	–5.62	0.2241	–5.47	0.0991
miR-132-3p	–0.62	–1.37	0.2381	–1.29	0.3511	–0.91	0.8370
miR-221-3p	–2.68	**−3.94**	**0.0316**	**−3.96**	**0.0370**	**−3.99**	**0.0150**
miR-10b	2.14	1.65	0.6900	**0.195**	0.0013*	1.10	0.0949
miR-212-3p	–0.48	–1.06	0.3908	–1.30	0.1647	–1.26	0.1364
miR-153-3p	–1.32	–2.25	0.0742	–1.80	0.5561	–1.99	0.2126
miR-101-5p	–1.66	–2.78	0.2891	–2.79	0.2913	–2.58	0.3736
miR-422a	–0.72	–1.25	0.6501	–1.41	0.4741	–1.83	0.0803
miR-23b-3p	–4.35	–4.71	0.8332	–5.01	0.4834	–5.33	0.1175
miR-133b	7.29	6.08	0.0686	6.32	0.2049	6.19	0.0772

ΔCTs of the 38 successfully amplified miRNAs are shown. P-values are from ANOVA with post-hoc Dunnett multiple comparison test between the disease and control groups. All bolded values are significant with α = 0.05. Asterisks (*) indicate p-values that are below Bonferroni correction value of 0.0013.

MiRNAs significantly upregulated across pathology groups are summarized in Table 3. Those altered in only one disease group included two (7%) miRNAs (miR-34a-5p and miR-10b-5p) upregulated in ALS; five miRNAs (18%; miR-124-3p, miR-181c-5p, miR-197-3p, miR-16-5p, and miR-100-5p) were significantly altered in CTE; and two (7%; miR-30c-5p and miR-210-3p) were unique to CTE + ALS. Of the miRNAs that were significantly altered in two disease groups, two miRNAs (7%; miR-146b-5p and miR-148a-3p) were upregulated in ALS and CTE; two (7%; miR-196a-5p and miR-30e-5p) were upregulated in both ALS and in the CTE + ALS; eleven (39%; miR-125b-5p, miR-9-5p, let-7i-5p, miR-26a-5p, miR-26b-5p, miR-30d-5p, miR-128-3p, miR-34c-5p, miR-19b-3p, miR-186 and let-7b-5p) were upregulated in CTE and CTE + ALS. Finally, four miRNAs (14%; miR-146a-5p, miR-107, miR-15a-5p and miR-221-3p) had significant upregulation in all 3 pathological groups (Figure 1). Only miR-10b had a p-value less than 0.00132 (Bonferroni corrected for multiple comparisons).

**TABLE 3:** Upregulated miRNAs between control and pathology groups.

MicroRNA	CTE	ALS	CTE+ALS
miR-107	✓	✓	✓
miR-181c-5p	✓		
miR-34c-5p	✓		✓
let-7b-5p	✓		✓
miR-9-5p	✓		✓
miR-125b-5p	✓		✓
miR-210-3p			✓
miR-124-3p	✓		
let-7d-5p			
miR-146b-5p	✓	✓	
miR-197-3p	✓		
miR-148a-3p	✓	✓	
miR-26b-5p	✓		✓
miR-26a-5p	✓		✓
miR-128-3p	✓		✓
miR-23a-3p			
miR-34a-5p		✓	
miR-100-5p	✓		
miR-16-5p	✓		
miR-19b-3p	✓		✓
miR-30d-5p	✓		✓
miR-30e-5p		✓	✓
let-7i-5p	✓		✓
miR-15a-5p	✓	✓	✓
miR-146a-5p	✓	✓	✓
miR-30c-5p			✓
miR-196a-5p		✓	✓
miR-186	✓		✓
miR-30a-5p			
miR-132-3p			
miR-221-3p	✓	✓	✓
miR-10b		✓	
miR-212-3p			
miR-153-3p			
miR-101-5p			
miR-422a			
miR-23b-3p			
miR-133b			

*✓ denotes if a miRNA ΔCT was upregulated in its pathological group in relation to the control group. Statistical analysis was done via ANOVA and Dunnett’s multiple comparison testing.*

**FIGURE 2:**
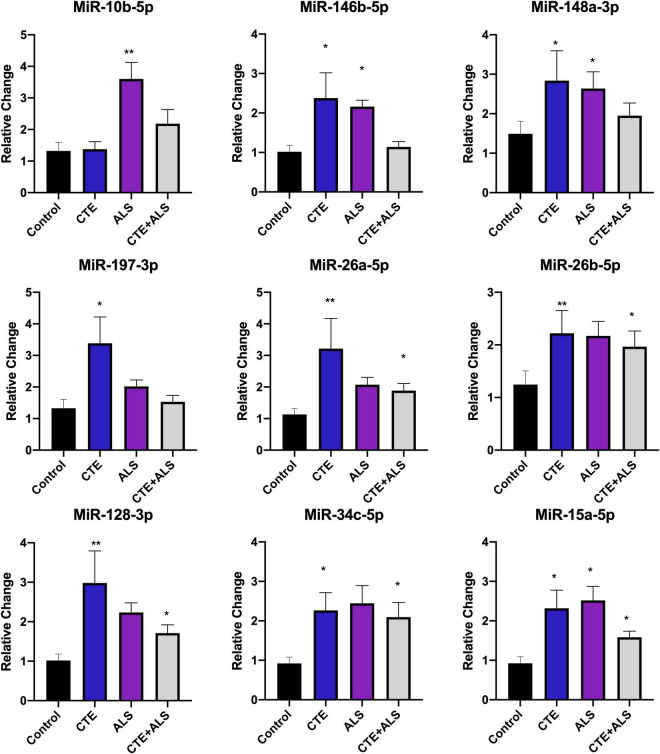
Cell Growth and Differentiation miRNAs significantly altered in CTE, ALS, or CTE + ALS. MiR-10b was upregulated in ALS alone. MiR-146b-5p and miR-148a-3p were significantly upregulated in non-comorbid ALS and CTE. MiR-197-3p was upregulated in CTE. Four miRNAs, miR-26a-5p, miR-26b-5p, miR-128-3p, miR-34c-5p were upregulated in CTE and CTE + ALS. Finally, miR-15a-5p was upregulated in all 3 conditions. Error bars denote standard error of the mean. *p < 0.05, **p < 0.01 compared to control group. Refer to Table 3 for statistical analyses between the pathologic groups and the control group.

**FIGURE 3:**
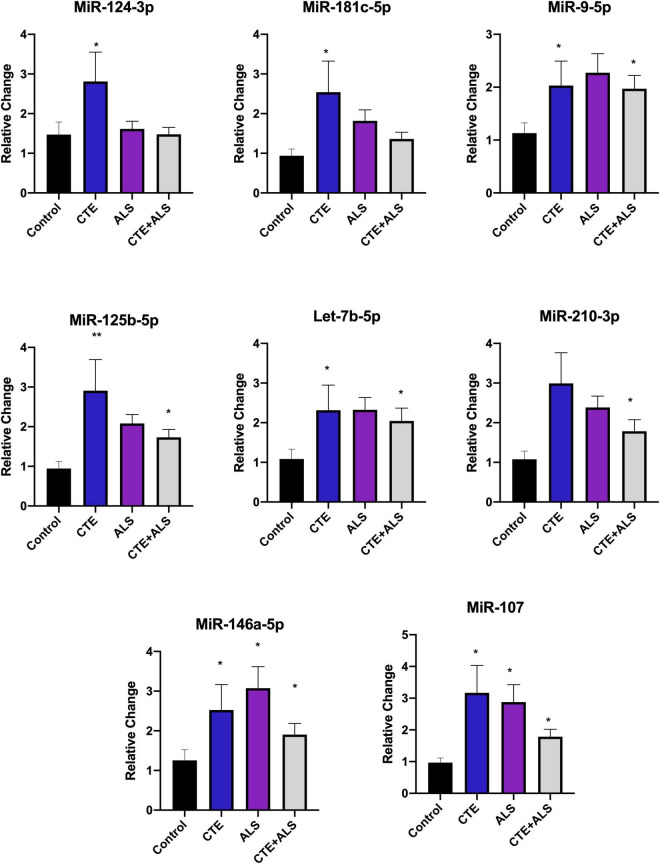
Inflammatory miRNAs significantly altered in CTE, ALS, or CTE + ALS. MiR-124-3p, miR-181c-5p were significantly upregulated in CTE only. MiR-9-5p, let-7b-5p, miR-125-5p, let-7b-5p were significantly upregulated in both CTE and CTE + ALS. MiR-210-3p was significantly upregulated in comorbid CTE + ALS. Finally, miR-146a-5p and miR-107 were significantly upregulated in all three groups. Error bars denote standard error of the mean. *p < 0.05, **p < 0.01 compared to control group. Refer to Table 3 for statistical analyses between the pathologic groups and the control group.

**FIGURE 4:**
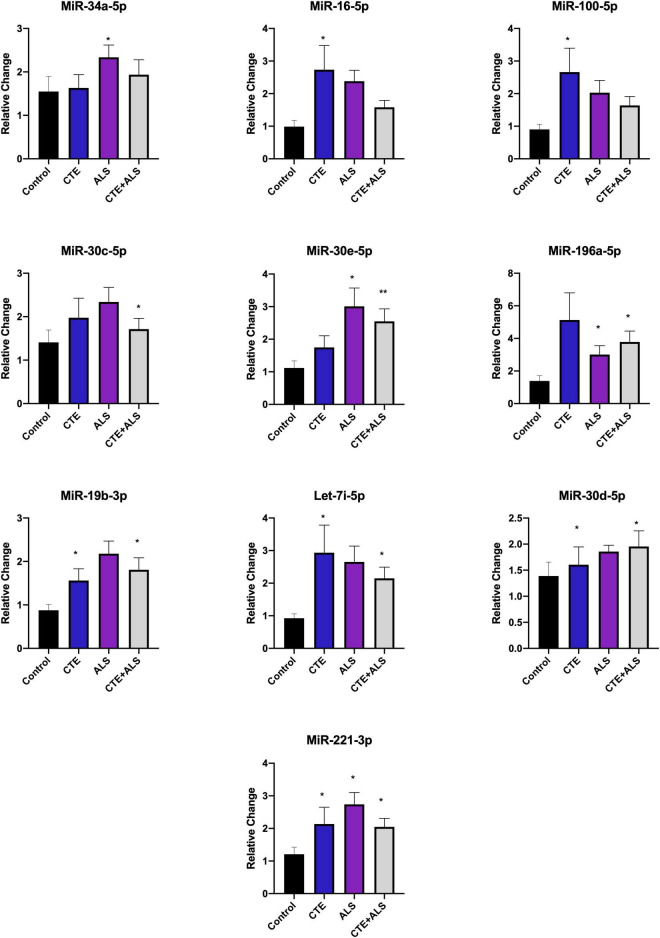
Apoptotic miRNAs significantly altered in CTE, ALS, or CTE + ALS. MiR-34a-5p-5p was significantly upregulated in ALS alone. miR-16-5p and miR-100-5p were upregulated in CTE. MiR-30c-5p was upregulated in comorbid CTE + ALS. MiR-196a-5p and miR-30e-5p miRNAs were upregulated in both ALS and CTE + ALS. Let-7i-5p, miR-30d-5p, and miR-19-3p were upregulated in both CTE and CTE + ALS. Finally, miR-221-3p was upregulated in all three groups. Error bars denote standard error of the mean. *p < 0.05, **p < 0.01 compared to control group. Refer to Table 3 for statistical analyses between the pathologic groups and the control group.

Based on previous studies, miRNAs were categorized according to their role in physiological processes. The majority of miRNAs altered in ALS, CTE, or CTE + ALS have roles in inflammation, apoptosis, or cell growth and differentiation (Supplementary Tables 3–5). Specifically, eight (29%) upregulated miRNAs are involved in inflammatory processes (Figure 2), nine (32%) are involved in cell growth and differentiation (Figure 3) and 10 (36%) play a role in apoptosis (Figure 4). There was one miRNA (3%) (miR-186) that was upregulated in CTE and CTE + ALS that has been shown to affect synaptic activity and inhibit BACE1 (Kim et al., 2016). The cell growth and differentiation miR-10b was increased in ALS, but not CTE or CTE + ALS (Figure 3). Apoptotic miRNAs were increased similarly across ALS, CTE, and CTE + ALS (Figure 4).

**FIGURE 5:**
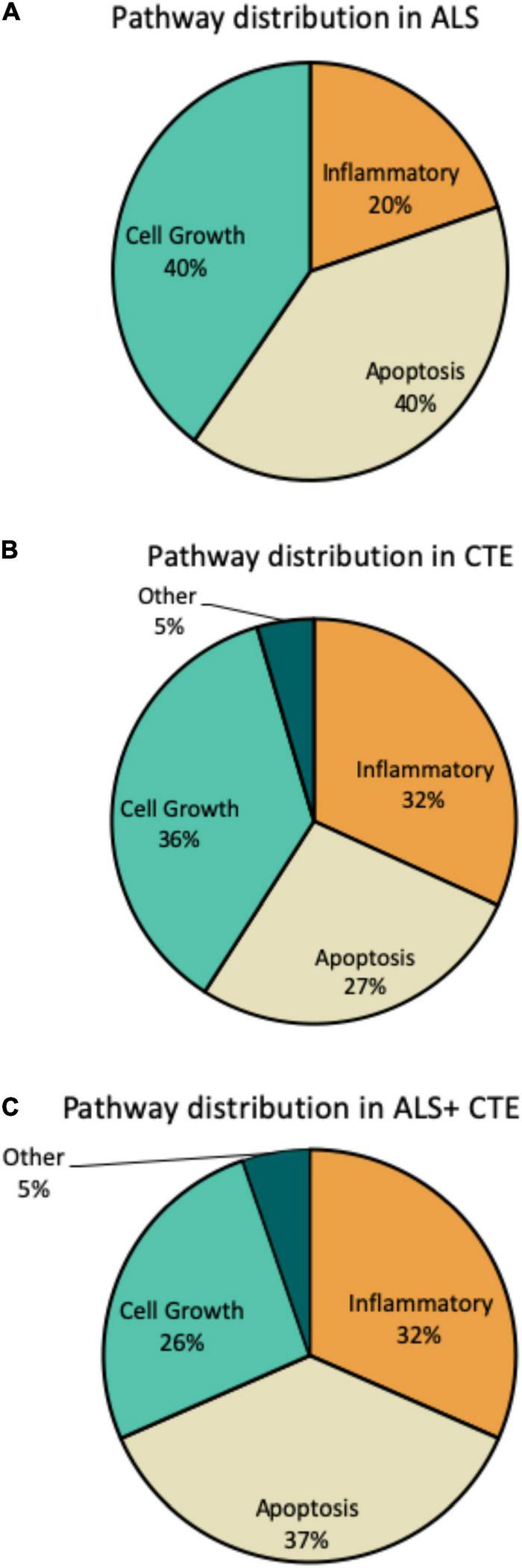
Frequency of miRNA pathways in disease. MiRNAs were classified into inflammatory, cell growth, apoptotic, and other pathways and the frequency within each pathway is shown. **(A)**. Frequency of miRNA pathways in ALS. **(B)**. Frequency of miRNA pathways in CTE. **(C)**. Frequency of miRNA pathways in CTE + ALS.

Within each disease, the percentage of miRNA pathways involved differed (Figure 5). In ALS, altered miRNAs were most frequently involved in cell growth (40%) and apoptosis (40%) and less frequently inflammation (20%). CTE also showed frequent alterations in cell growth (36%), but greater involvement in inflammatory pathways (32%) compared to ALS. Finally, when ALS and CTE were comorbid, apoptosis (37%) and inflammatory (32%) pathways were the most frequently involved.

## Discussion

Overall, we found that CTE and ALS were characterized by similar changes in miRNAs previously implicated in neurological disease. The majority of miRNAs (72%) were similarly involved in ALS and CTE, suggesting common pathogenetic pathways of inflammation, cell growth, and apoptosis. The most significantly changed miRNA was miR-10b-5p, which was increased in ALS.

### Cell Growth and Differentiation Pathways

MiR-10b is involved in cell growth and differentiation pathways. Mir-10b-5p has been shown to interact with the HOX gene cluster in both Alzheimer’s disease (AD) (Ruan et al., 2021) and Huntington’s disease (Hoss et al., 2014). In Alzheimer disease Ruan and colleagues showed that HOX genes were decreased and inhibited by miR-10b-5p, leading to more severe disease. Hoss et al. showed that three miRNAs that originate at or near the HOX gene cluster, miR-10b-5p, miR-196a-5p and miR-148a-3p, are significantly upregulated in Huntington’s disease, a neurodegeneration characterized by motor dysfunction, personality change, and cognitive decline. MiR-10b-5p has been studied in its relation to ALS though results have been mixed. Down regulation in ALS has been observed in muscle tissue (Si et al., 2018) and in plasma (Banack et al., 2020), but upregulation has been observed in whole blood (De Felice et al., 2018).

Another target of miR-10b-5p is brain derived neurotrophic growth factor (BDNF), which is a key regulator of cell growth and plasticity in the brain and has been shown to enhance cell survival. MiR-10b-5p has been shown to directly inhibit BDNF (L. Wang et al., 2020). The BDNF/TrkB pathway has been shown to be altered in ALS and BDNF was increased in skeletal muscle (Lanuza et al., 2019). Decreases in BDNF have also been reported after TBI (Korley et al., 2016), in AD, and in aging (Jiao et al., 2016). Other miRNAs that disrupt BDNF and may be involved include miR-26a-5p, miR-26b-5p, and miR-15a-5p. These were also found to be altered in CTE and CTE + ALS.

### Inflammatory Pathways

Altered inflammatory pathways are a feature of RHI and CTE (Cherry et al., 2016, 2021) and ALS (Spencer et al., 2020), and numerous miRNAs might regulate these processes (Supplementary Table 3). Of the miRNAs altered in CTE and CTE + ALS compared to ALS, many were inflammatory, which supports the roles of RHI and inflammation in CTE pathogenesis. On the other hand, some inflammatory miRNAs were upregulated similarly in all three disease groups (miR-146a-5p, miR-107). MiR-146a-5p and miR-107 along with miR-9-5p, miR-181c-5p and miR-125b-5p are involved in the NF-κB pathway. The NF-κB has been previously implicated in ALS (Parisi et al., 2016; Tahamtan et al., 2018; Slota and Booth, 2019; Källstig et al., 2021) and TBI (Jassam et al., 2017; Pierre et al., 2021). Outside of the NF-κB pathway, miR-125b-5p has been directly implicated in hyperphosphorylation of tau (Banzhaf-Strathmann et al., 2014) and might contribute to CTE pathogenesis.

### Apoptotic Pathways

Several upregulated miRNAs have a role in apoptosis and autophagy in neurodegenerative diseases. Protein and damaged cell clearance are especially important in ALS and CTE in which abnormal p-TDP-43 and p-tau proteins accumulate. MiRNAs related to apoptosis and autophagy were the predominantly altered group in ALS and CTE + ALS (Figure 5). MiR-34a-5p upregulation was unique to ALS. It has been previously demonstrated an upregulation in the plasma of familial ALS research participants with the C9orf72 mutation (Kmetzsch et al., 2021). The autophagy pathway is primarily regulated by inhibition of mTOR (mammalian target of rapamycin). mTOR is directly inhibited by miR-100-5p which was also found to be upregulated in CTE and has previously been implicated in altered protein deposition in AD (Ye et al., 2015). PI3K/akt, an activator of mTOR which is inhibited by miR-16-5p (T. Li et al., 2019), also found to be upregulated in CTE. Another key player in both apoptosis and autophagy pathways is Beclin, which fosters removal of old proteins and damaged cells. MiR-30c-5p, miR-30d-5p, and miR-30e-5p were all upregulated in ALS and CTE and have been shown to inhibit Beclin (Millan, 2017; Zhao et al., 2017), a protein involved in apoptosis and autophagy.

### Biomarker Development

MiRNAs have been proposed as potential biomarkers for disease (Ghosh et al., 2021). Currently, CTE can only be diagnosed at autopsy and ALS is typically diagnosed after motor functions have declined. It remains to be determined whether miRNAs, such as miR-10b-5p are altered in biofluids such as the cerebrospinal fluid or blood during life in individuals with ALS or CTE. There have been recent studies that have examined the utility of select miRNA biomarkers in blood. MiR-181 is widely expressed in neurons and may be a marker for neuronal density, and miR-181 levels in serum have recently been associated with increased risk of death in ALS (Magen et al., 2021). We did not see differences in ALS prefrontal cortex but found that miR-181c-5p was significantly upregulated in the CTE group. Other promising miRNA targets that may be used as blood biomarkers of ALS patients include miR-206 and miR-124-3p (Soliman et al., 2021; Vaz et al., 2021). Correlations with blood and brain miRNA levels require further study.

### Limitations

There were several limitations to this study. Only select miRNAs previously implicated in neurological disease were tested. Future studies should include more cases and examine additional miRNA targets as well as correlation between miRNA expression and markers for inflammation, cell growth, and apoptosis. This study also focused on changes in miRNAs within postmortem tissue from prefrontal cortex. Whether these changes are specific to prefrontal cortex in CTE and ALS or characteristics of widespread brain areas remains to be determined. Postmortem human brain tissue was evaluated; however, miRNAs are generally stable to degradation, and RIN values, a measure of tissue quality, were not significantly different between groups. Study participants were limited to primarily Caucasian men, limiting the generalizability of these findings.

## Conclusion

Shared miRNA alterations in CTE and ALS suggest that inflammation, apoptosis, and cell growth are neurodegenerative pathways common to both disorders. Unique increases in miR-100-5p in brain donors with CTE, and unique increases in miR-10b-5p in brain donors with ALS suggest that miRNA analysis might prove useful in distinguishing these disorders but will require future studies of additional brain donors using broader regions of brain and spinal cord. Future studies in biofluids during life are warranted, including cerebrospinal fluid and serum, to determine the utility of miRNAs for potential biomarker development. Overall, these shared and distinct miRNA profiles suggest that miRNA analysis might prove useful in the future development of biomarkers for CTE and ALS.

## Data Availability Statement

The original contributions presented in the study are included in the article/Supplementary Material, further inquiries can be directed to the corresponding author.

## Author Contributions

MaA and TS: study design, conception, and drafting of the manuscript. MaA, TS, NA, KS, ZF, NR, LG, IR, JA, SW, VA, BH, RM, KC, RN, MP, AL, FA, JM, NK, AM, and CB: acquisition and analysis of data. All authors contributed to the article and approved the submitted version.

## Conflict of Interest

The authors declare that the research was conducted in the absence of any commercial or financial relationships that could be construed as a potential conflict of interest.

## Publisher’s Note

All claims expressed in this article are solely those of the authors and do not necessarily represent those of their affiliated organizations, or those of the publisher, the editors and the reviewers. Any product that may be evaluated in this article, or claim that may be made by its manufacturer, is not guaranteed or endorsed by the publisher.
