# Supplementary material for: MicroRNA Alterations in Chronic Traumatic Encephalopathy and Amyotrophic Lateral Sclerosis
Source: Front Neurosci. 2022 May 19;16:855096. doi: 10.3389/fnins.2022.855096 (PMC9160996; doi:10.3389/fnins.2022.855096)
Supplement: Supplementary file 1 [file Table_1.docx]

**S.1 - MicroRNA Assay IDs**

| Number | MiRNA | Assay Name | Assay ID |
| --- | --- | --- | --- |
| 1 | miRNA-423 | hsa-miR-423-3p | 478327_mir |
| 2 | MiRNA-155 | hsa-miR-155-5p | 483064_mir |
| 3 | MiRNA-146a | hsa-miR-146a-5p | [478399_mir](https://www.thermofisher.com/order/genome-database/details/microrna/478399_mir?CID=&ICID=&subtype=) |
| 4 | MiRNA-146b | hsa-miR-146b-5p | 483144_mir |
| 5 | MiRNA-125b | hsa-miR-125b-5p | [477885_mir](https://www.thermofisher.com/order/genome-database/details/microrna/477885_mir?CID=&ICID=&subtype=) |
| 6 | MiRNA-9 | hsa-miR-9-5p | [478214_mir](https://www.thermofisher.com/order/genome-database/details/microrna/478214_mir?CID=&ICID=&subtype=) |
| 7 | MiRNA-30a | hsa-miR-30a-5p | [479448_mir](https://www.thermofisher.com/order/genome-database/details/microrna/479448_mir?CID=&ICID=&subtype=) |
| 8 | MiRNA-30b | hsa-miR-30b-3p | [478804_mir](https://www.thermofisher.com/order/genome-database/details/microrna/478804_mir?CID=&ICID=&subtype=) |
| 9 | MiRNA-30c | hsa-miR-30c-5p | [478008_mir](https://www.thermofisher.com/order/genome-database/details/microrna/478008_mir?CID=&ICID=&subtype=) |
| 10 | MiRNA-30e | hsa-miR-30e-5p | [479235_mir](https://www.thermofisher.com/order/genome-database/details/microrna/479235_mir?CID=&ICID=&subtype=) |
| 11 | MiRNA-30d | hsa-miR-30d-5p | [478606_mir](https://www.thermofisher.com/order/genome-database/details/microrna/478606_mir?CID=&ICID=&subtype=) |
| 12 | MiRNA-132 | hsa-miR-132-3p | [477900_mir](https://www.thermofisher.com/order/genome-database/details/microrna/477900_mir?CID=&ICID=&subtype=) |
| 13 | MiRNA-206 | hsa-miR-206 | [477968_mir](https://www.thermofisher.com/order/genome-database/details/microrna/477968_mir?CID=&ICID=&subtype=) |
| 14 | MiRNA-29 | hsa-miR-29b-1-5p | [478794_mir](https://www.thermofisher.com/order/genome-database/details/microrna/478794_mir?CID=&ICID=&subtype=) |
| 15 | MiRNA-124a | hsa-miR-124-3p | [480901_mir](https://www.thermofisher.com/order/genome-database/details/microrna/480901_mir?CID=&ICID=&subtype=) |
| 16 | MiRNA-128 | hsa-miR-128-3p | [477892_mir](https://www.thermofisher.com/order/genome-database/details/microrna/477892_mir?CID=&ICID=&subtype=) |
| 17 | MiRNA-107 | hsa-miR-107 | [478254_mir](https://www.thermofisher.com/order/genome-database/details/microrna/478254_mir?CID=&ICID=&subtype=) |
| 18 | MiRNA-34a | hsa-miR-34a-5p | [478048_mir](https://www.thermofisher.com/order/genome-database/details/microrna/478048_mir?CID=&ICID=&subtype=) |
| 19 | MiRNA-34b | hsa-miR-34b-5p | [478050_mir](https://www.thermofisher.com/order/genome-database/details/microrna/478050_mir?CID=&ICID=&subtype=) |
| 20 | MiRNA-34c | hsa-miR-34c-5p | [478052_mir](https://www.thermofisher.com/order/genome-database/details/microrna/478052_mir?CID=&ICID=&subtype=) |
| 21 | let-7i | hsa-let-7i-5p | [478375_mir](https://www.thermofisher.com/order/genome-database/details/microrna/478375_mir?CID=&ICID=&subtype=) |
| 22 | MiRNA-221 | hsa-miR-221-3p | [477981_mir](https://www.thermofisher.com/order/genome-database/details/microrna/477981_mir?CID=&ICID=&subtype=) |
| 23 | MiRNA-222 | hsa-miR-222-3p | [478779_mir](https://www.thermofisher.com/order/genome-database/details/microrna/478779_mir?CID=&ICID=&subtype=) |
| 24 | MiRNA-10b | hsa-miR-10b-5p | [478494_mir](https://www.thermofisher.com/order/genome-database/details/microrna/478494_mir?CID=&ICID=&subtype=) |
| 25 | MiRNA-196a | hsa-miR-196a-5p | [478230_mir](https://www.thermofisher.com/order/genome-database/details/microrna/478230_mir?CID=&ICID=&subtype=) |
| 26 | MiRNA-196b | hsa-miR-196b-5p | [478585_mir](https://www.thermofisher.com/order/genome-database/details/microrna/478585_mir?CID=&ICID=&subtype=) |
| 27 | MiRNA-615 | hsa-miR-615-3p | [478175_mir](https://www.thermofisher.com/order/genome-database/details/microrna/478175_mir?CID=&ICID=&subtype=) |
| 28 | MiRNA-133b | hsa-miR-133b | [480871_mir](https://www.thermofisher.com/order/genome-database/details/microrna/480871_mir?CID=&ICID=&subtype=) |
| 29 | MiRNA-26a | hsa-miR-26a-5p | 477995_mir |
| 30 | MiRNA-26b | hsa-miR-26b-5p | [478418_mir](https://www.thermofisher.com/order/genome-database/details/microrna/478418_mir?CID=&ICID=&subtype=) |
| 31 | MiRNA-181c | hsa-miR-181c-5p | 477934_mir |
| 32 | MiRNA-212 | hsa-miR-212-3p | [478318_mir](https://www.thermofisher.com/order/genome-database/details/microrna/478318_mir?CID=&ICID=&subtype=) |
| 33 | MiRNA-153 | hsa-miR-153-3p | [477922_mir](https://www.thermofisher.com/order/genome-database/details/microrna/477922_mir?CID=&ICID=&subtype=) |
| 34 | MiRNA-101 | hsa-miR-101-5p | [478620_mir](https://www.thermofisher.com/order/genome-database/details/microrna/478620_mir?CID=&ICID=&subtype=) |
| 35 | MiRNA-210 | hsa-miR-210-3p | [477970_mir](https://www.thermofisher.com/order/genome-database/details/microrna/477970_mir?CID=&ICID=&subtype=) |
| 36 | MiRNA-19b | hsa-miR-19b-3p | [478264_mir](https://www.thermofisher.com/order/genome-database/details/microrna/478264_mir?CID=&ICID=&subtype=) |
| 37 | MiRNA-197 | hsa-miR-197-3p | [477959_mir](https://www.thermofisher.com/order/genome-database/details/microrna/477959_mir?CID=&ICID=&subtype=) |
| 38 | MiRNA-15a | hsa-miR-15a-5p | [477858_mir](https://www.thermofisher.com/order/genome-database/details/microrna/477858_mir?CID=&ICID=&subtype=) |
| 39 | MiRNA-16 | hsa-miR-16-5p | [477860_mir](https://www.thermofisher.com/order/genome-database/details/microrna/477860_mir?CID=&ICID=&subtype=) |
| 40 | MiRNA-186 | hsa-miR-186-5p | [477940_mir](https://www.thermofisher.com/order/genome-database/details/microrna/477940_mir?CID=&ICID=&subtype=) |
| 41 | MiRNA-100 | hsa-miR-100-5p | [478224_mir](https://www.thermofisher.com/order/genome-database/details/microrna/478224_mir?CID=&ICID=&subtype=) |
| 42 | MiRNA-144 | hsa-miR-144-5p | [477914_mir](https://www.thermofisher.com/order/genome-database/details/microrna/477914_mir?CID=&ICID=&subtype=) |
| 43 | MiRNA-422a | hsa-miR-422a | [478481_mir](https://www.thermofisher.com/order/genome-database/details/microrna/478481_mir?CID=&ICID=&subtype=) |
| 44 | MiRNA-148a | hsa-miR-148a-3p | [477814_mir](https://www.thermofisher.com/order/genome-database/details/microrna/477814_mir?CID=&ICID=&subtype=) |
| 45 | MiRNA-23a | hsa-miR-23a-3p | [478532_mir](https://www.thermofisher.com/order/genome-database/details/microrna/478532_mir?CID=&ICID=&subtype=) |
| 46 | MiRNA-23b | hsa-miR-23b-3p | [483150_mir](https://www.thermofisher.com/order/genome-database/details/microrna/483150_mir?CID=&ICID=&subtype=) |
| 47 | let-7b | hsa-let-7b-5p | 478576_mir |
| 48 | let-7d | hsa-let-7d-5p | [478439_mir](https://www.thermofisher.com/order/genome-database/details/microrna/478439_mir?CID=&ICID=&subtype=) |
