# Supplementary material for: MicroRNA Alterations in Chronic Traumatic Encephalopathy and Amyotrophic Lateral Sclerosis
Source: Front Neurosci. 2022 May 19;16:855096. doi: 10.3389/fnins.2022.855096 (PMC9160996; doi:10.3389/fnins.2022.855096)
Supplement: Supplementary file 2 [file Table_2.docx]

**S.2- miRNA Implicated in the Brains of Humans with Neurodegenerative Diseases**

| MicroRNA | Disease Observed | Region Tested | Up/Down Regulation | Sources |
| --- | --- | --- | --- | --- |
| MiRNA-155-5p | ALS | Spinal Cord, Cerebral Spinal Fluid | Up | (Butovsky et al., 2015) |
|  | AD | Temporal Lobe, Neocortex | Up | (Lukiw et al., 2013) |
|  | PD | Blood | Up | (Caggiu et al., 2018) |
| MiRNA-146a-5p | AD | Temporal Lobe | Up | (Cui et al., 2010) |
|  | ALS | Spinal Cord | Up | (Campos-Melo et al., 2013) |
| MiRNA-146b-5p | AD | Hippocampus, Frontal Cortex Temporal Cortex Cerebral Spinal Fluid | Down | (Millan, 2017) |
| MiRNA-125b-5p | ALS | Spinal Cord | Up | (Parisi et al., 2016) |
|  | AD | Frontal Cortex, Hippocampus | Up | (Ma et al., 2017) |
|  | PD | Plasma | Down | (L. Chen et al., 2018) |
| MiRNA-9-5p | ALS | Hippocampus, Brainstem | Down Hippocampus  Up in Brainstem | (Marcuzzo et al., 2015) |
|  | AD | Frontal Gyrus | Down | (Sethi & Lukiw, 2009) |
| MiRNA-30b-3p | MS/ALS/AD/PD | Cerebral Spinal Fluid | Up | (Brennan et al., 2019) |
| MiRNA-30b-3p  MiRNA-30c-5p  MiRNA-30e-5p | HD | Frontal Cortex, Striatum | Up | (Martí et al., 2010) |
| MiRNA-30a-5p  MiRNA-30b-3p  MiRNA-30c-5p  MiRNA-30d-5p  MiRNA-30e-5p | AD | Frontal Cortex Temporal Cortex Cerebral Spinal Fluid, Hippocampus, Parietal Cortex | UP in Frontal Cortex and Cerebral Spinal Fluid  Down in Temporal Cortex and Parietal Cortex | (Millan, 2017) |
| MiRNA-132-3p | AD | Temporal Cortex, Hippocampus | Down | (Wong et al., 2013) |
|  |  | Cerebral Spinal Fluid | Up | (Gui et al., 2015) |
| MiRNA-206 | ALS | Circulating, Muscles | Up | (Toivonen et al., 2014) |
|  | AD | Temporal Cortex | Up | (Lee et al., 2012) |
| MiRNA-29b-5p | AD | Cerebral Spinal Fluid | Up in Cerebral Spinal Fluid Down in Cortex | (Hébert et al., 2008) |
| MiRNA-124-3p | AD | Hippocampus | Down | (Sun et al., 2015) |
|  | ALS | Leukocytes | Up | (Vrabec et al., 2018) |
| MiRNA-128-3p | AD | Hippocampus, | Up | (Adlakha & Saini, 2014) |
|  | ALS | Circulating | Down | (Liguori et al., 2018) |
| MiRNA-107 | AD | Superior and Middle Temporal Cortex, Motor Cortex | Down | (W. X. Wang et al., 2008) |
| MiRNA-34a-5p | AD | Hippocampus, | Up | (Basavaraju & De Lencastre, 2016) |
| MiRNA-34b-5p  MiRNA-34c-5p | PD | Frontal Cortex, Substantia Nigra | Down | (Karnati et al., 2015) |
| let-7i-5p | AD | Cortex | Up | (Karnati et al., 2015) |
|  | PD | Frontal Cortex | Down | (Martí et al., 2010) |
| MiRNA-221-3p | HD | Frontal Cortex | Down | (Martí et al., 2010) |
|  | ALS | Muscle | Down | (Pegoraro et al., 2017) |
| MiRNA-222-3p | HD | Frontal Cortex | Down | (Martí et al., 2010) |
|  | PD | Serum | Down | (L. Chen et al., 2018) |
| MiRNA-10b-5p | HD | Pre-Frontal Cortex | Up | (Hoss et al., 2014) |
| MiRNA-196a-5p | HD | Pre-Frontal Cortex | Up | (Hoss et al., 2014) |
| MiRNA-196b-5p | HD | Pre-Frontal Cortex | Up | (Hoss et al., 2014) |
|  | AD | Blood | Up | (Kamal & Place, 2018) |
| MiRNA-615-3p | HD | Pre-Frontal Cortex | Up | (Hoss et al., 2014) |
| MiRNA-133b | PD | Midbrain | Down | (Kamal & Place, 2018)  (Mushtaq et al., 2016) |
|  | ALS | Blood | Up | (Ricci et al., 2018) |
| MiRNA-26a-5p | AD | Temporal Cortex, Hippocampus | Up | (Absalon et al., 2013) |
|  | ALS | Skeletal Muscle | Down | (Jensen et al., 2016) |
|  | PD | Cerebral Spinal Fluid | Down | (Su et al., 2019) |
| MiRNA-26b-5p | AD | Temporal Cortex, Hippocampus | Up | (Absalon et al., 2013) |
| MiRNA-181c-5p | AD | Temporal Cortex, Serum, Parietal Cortex Frontal Cortex | Down | (Femminella et al., 2015) |
| MiRNA-212-3p | AD | Temporal Cortex, Hippocampus, Frontal Cortex | Down | (Wong et al., 2013) |
|  |  |  |  |  |
| MiRNA-153-3p | AD | Frontal Cortex | Down | (Long et al., 2012) |
| MiRNA-101-5p | AD | Anterior Temporal Cortex | Down | (Hébert et al., 2008) |
| MiRNA-210-3p | AD | Anterior Temporal Cortex | Down | (Hébert et al., 2008) |
| MiRNA-19b-3p | AD | Anterior Temporal Cortex | Down | (Hébert et al., 2008) |
|  | PD | Substantia Nigra | Down | (Taguchi & Wang, 2018) |
| MiRNA-197-3p | AD | Anterior Temporal Cortex | Down | (Hébert et al., 2008) |
| MiRNA-15a-5p | AD | Anterior Temporal Cortex | Down | (Hébert et al., 2008) |
| MiRNA-16-5p | AD | Hippocampus, Cerebral Spinal Fluid | Down | (Millan, 2017) |
|  | ALS | Spinal Cord | Up | (Campos-Melo et al., 2013) |
|  |  | Blood | Down | (Liguori et al., 2018) |
| MiRNA-186-5p | AD | Frontal Cortex, Temporal Cortex Cerebral Spinal Fluid | Increase in Frontal Cortex and Cerebral Spinal Fluid, Down Temporal Cortex | (Millan, 2017) |
| MiRNA-100-5p | AD | Frontal Cortex, Cerebral Spinal Fluid, | Up | (Millan, 2017) |
| MiRNA-144-5p | AD | Temporal Cortex, Frontal Cortex | Up in Frontal Cortex Down In Temporal Cortex | (Millan, 2017) |
|  | ALS | Whole Blood/Serum | Up in Serum  Down in Whole Blood | (Ricci et al., 2018) |
|  | PD | Anterior Cingulate Gyri, Frontal Cortex | Up in Anterior Cingulate Gyri  Down in Frontal Cortex | Tatura et al., 2016, Thomas et al., 2012 |
| MiRNA-422a | AD | Hippocampus, Frontal Cortex Temporal cortex | Down In Temporal Cortex Up In Hippocampus And Frontal Cortex | (Millan, 2017) |
|  | MS | Serum | Up | (C. Chen et al., 2018) |
| MiRNA-148a-3p | AD | Frontal Cortex, Temporal Cortex, Parietal Cortex | Up in Frontal Cortex Down in Temporal and Parietal Cortex | (Millan, 2017) |
| MiRNA-148a-3p | ALS | Cerebral Spinal Fluid | Up | (Joilin et al., 2019) |
| MiRNA-23a-3p  MiRNA-23b-5p | AD | Hippocampus Frontal Cortex | Up in Hippocampus Down in Frontal Cortex | (Millan, 2017) |
|  | ALS | Skeletal Muscle | Up | (Russell et al., 2013) |
| let-7b-5p | AD | Substantia Nigra | Down | (Karnati et al., 2015) |
|  | ALS | Blood | Up | (Vrabec et al., 2018) |
| let-7d-5p | AD | Frontal Cortex | Down | (Weinberg et al., 2015) |
|  | ALS | Blood | Down | (Ricci et al., 2018) |

Supplementary References

Butovsky, O., Jedrychowski, M. P., Cialic, R., Krasemann, S., Murugaiyan, G., Fanek, Z., et al. (2015). Targeting miR-155 restores abnormal microglia and attenuates disease in SOD1 mice: role of miR-155 in ALS. Ann. Neurol. 77, 75–99. doi: 10.1002/ana.24304

Hebert, S. S., Horre, K., Nicolai, L., Papadopoulou, A. S., Mandemakers, W., Silahtaroglu, A. N., et al. (2008). Loss of microRNA cluster miR-29a/b-1 in sporadic Alzheimer’s disease correlates with increased BACE1/ -secretase expression. Proc. Natl. Acad. Sci. U.S.A. 105, 6415–6420. doi: 10.1073/pnas.0710263105

Mushtaq, Gohar, Nigel H. Greig, Firoz Anwar, Mazin A. Zamzami, Hani Choudhry, Munvar M. Shaik, Ian A. Tamargo, and Mohammad A. Kamal. “MiRNAs as Circulating Biomarkers for Alzheimer’s Disease and Parkinson’s Disease.” *Medicinal Chemistry* 12, no. 3 (March 31, 2016): 217–25. https://doi.org/10.2174/1573406411666151030112140.
