# Supplementary material for: MicroRNA Alterations in Chronic Traumatic Encephalopathy and Amyotrophic Lateral Sclerosis
Source: Front Neurosci. 2022 May 19;16:855096. doi: 10.3389/fnins.2022.855096 (PMC9160996; doi:10.3389/fnins.2022.855096)
Supplement: Supplementary file 3 [file Table_3.docx]

**S.3 –MiRNAs implicated in cell growth and differentiation**

| MiRNA | Significant Expression | Cell Growth/Differentiation Component | Citation |
| --- | --- | --- | --- |
| miR-146b-5p | ALS, CTE | Smad4 | (N. Zhang et al., 2017) |
| miR-34c-5p | CTE, CTE+ALS | Sirt1 | (Kao et al., 2018) |
| miR-148a-3p | ALS, CTE | TGF-β/Smad | (H. Wang et al., 2015) |
| miR-128-3p | CTE, CTE+ALS | PCM1 | (W. Zhang et al., 2016) |
| miR-26b-5p | CTE, CTE+ALS | BDNF | (Caputo et al., 2011) |
| miR-26a-5p | CTE, CTE+ALS | BDNF | (Caputo et al., 2011) |
| miR-197-3p | CTE | ADAM10  NOTCH | (Y.-M. Wang et al., 2019) |
| MiR-15a-5p | ALS, CTE, CTE+ALS | BDNF | (Gao et al., 2015) |
| miR-10b-5p | ALS | BDNF | (L. Wang et al., 2020) |
