# Supplementary material for: MicroRNA Alterations in Chronic Traumatic Encephalopathy and Amyotrophic Lateral Sclerosis
Source: Front Neurosci. 2022 May 19;16:855096. doi: 10.3389/fnins.2022.855096 (PMC9160996; doi:10.3389/fnins.2022.855096)
Supplement: Supplementary file 4 [file Table_4.docx]

**S.4 –MiRNAs implicated in inflammatory pathways**

| MiRNA | Significant Expression | Inflammatory Component | Citation |
| --- | --- | --- | --- |
| miR-9-5p | CTE, ALS + CTE | IL-1β, IL-6, and MCP-1, NF-κB | (Tahamtan et al., 2018) |
| miR-107 | ALS, CTE, ALS+ CTE | Progranulin | (Chitramuthu et al., 2017) |
| Let-7b-5p | CTE ALS+ CTE | TLR-4, C/EBP-Sigma, IL-6, IL-10 | (Slota & Booth, 2019) |
| miR-181c-5p | CTE | NF-κB | (Tahamtan et al., 2018) |
| miR-125b-5p | CTE, CTE+ALS | A20 (NF-κB pathway) | (Parisi et al., 2016) |
| miR-210-3p | ALS + CTE | Sirt1 | (B. Li et al., 2020) |
| miR-124-3p | CTE, CTE+ALS | MyD88, TLR-6 C/EBP-α, Sirt1 | (Slota & Booth, 2019), (Heyn et al., 2016) |
| MiR-146a-5p | ALS, CTE, CTE+ALS | NF-κB, IRAK1, TRAF6,  STAT-1 | (Slota & Booth, 2019) |
