# Supplementary material for: MicroRNA Alterations in Chronic Traumatic Encephalopathy and Amyotrophic Lateral Sclerosis
Source: Front Neurosci. 2022 May 19;16:855096. doi: 10.3389/fnins.2022.855096 (PMC9160996; doi:10.3389/fnins.2022.855096)
Supplement: Supplementary file 5 [file Table_5.docx]

**S.5 –MiRNAs implicated in apoptotic pathways**

| MiRNA | Significant Expression | Apoptotic Component | Citation |
| --- | --- | --- | --- |
| miR-30e-5p | ALS, CTE+ALS | Beclin1 | (Millan, 2017) |
| miR-30d-5p | CTE, CTE+ALS | Beclin1 | (F. Zhao et al., 2017) |
| miR-19b-3p | CTE, CTE+ALS | Pten, SOX6 | (Marcuzzo et al., 2015) |
| miR-30c-5p | CTE+ALS | Xbp1,TGF-ß, Beclin1 | (Millan, 2017) |
| miR-16-5p | CTE | (MAPK) and PI3K/Akt | (T. Li et al., 2019) |
| miR-34a-5p | ALS | P53 | (Guessous et al., 2010) |
| let-7i-5p | ALS, CTE, CTE+ALS | Ubiquitin C-terminal hydrolase L1, TNF- α | (Balakathiresan et al., 2012) |
| miR-196a-5p | ALS, CTE, CTE+ALS | ANXA1 | (C. Chen et al., 2011) |
| miR-221-3p | ALS, CTE, CTE+ALS | Pten | (Peng et al., 2020) |
| miR-100-5p | CTE | mTOR | (Ye et al., 2015) |
